# First Annual Report of the Registrar-General of Births, &c. in England

**Published:** 1839-10

**Authors:** 


Art. III.—*First Annual Report of the Registrar-General of Births, Deaths, and Marriages, in England.* — *London,* 1839. 8vo, pp. 168.

We regret exceedingly that we are unable to notice this most important document in our present Number with that minuteness of detail which it requires and deserves. We hope to do this in our next publication ; but we cannot allow the present to come before our readers unaccompanied with the expression of our opinion as to the admirable manner in which the Report has been drawn up, and our earnest recommendation of its contents to every member of the profession. We think no one can read it without being strongly impressed with the vast advantages to medical science and to human happiness which may confidently be expected from the Registration Act of which this Report is the first-fruits: and we are quite sure that no right-minded member of the profession can hesitate for a moment to promote the successful working of this Act by giving every facility to the furtherance of its characteristic and most important feature, the registration of the exact causes of death. By the publication of this Report, the registrar-general (Mr. Lister) has demonstrated his peculiar fitness for the high office which he fills. He seems duly impressed with the importance of its duties, and displays a comprehensiveness of view, a clearness of method, and an industrious zeal, which cannot fail to lead to great results. In no point, however, does he appear to us to have shown more judgment than in the appointment of Mr. Farr to draw up the medical portion of the Report. His "Letter to the Registrar-General, with Abstracts of the recorded causes of 141,607 Deaths," is such as the profession were prepared to expect from the acknowledged preeminence of that gentleman in the science of vital statistics.

